# Structural and electronic properties of Eu- and Pd-doped ZnO

**DOI:** 10.1186/1556-276X-6-357

**Published:** 2011-04-21

**Authors:** Mohammad Hussein Naseef Assadi, Yuebin Zhang, Rong-Kun Zheng, Simon Peter Ringer, Sean Li

**Affiliations:** 1 Australian Centre for Microscopy and Microanalysis, The University of Sydney, Sydney, NSW 2006, Australia; 2 School of Materials Science and Engineering, The University of New South Wales, Sydney, NSW 2052, Australia

## Abstract

Doping ZnO with rare earth and 4d transition elements is a popular technique to manipulate the optical properties of ZnO systems. These systems may also possess intrinsic ferromagnetism due to their magnetic moment borne on 4*f *and 4*d *electrons. In this work, the structural, electronic, and magnetic properties of Eu- and Pd-doped ZnO were investigated by the *ab initio *density functional theory methods based on generalized gradient approximation. The relative stability of incorporation sites of the doped elements in the ZnO host lattice was studied. The ground state properties, equilibrium bond lengths, and band structures of both the ZnO:Eu and ZnO:Pd systems were also investigated. The total and partial densities of electron states were also determined for both systems. It was found that in the ZnO:Eu system, ambient ferromagnetism can be induced by introducing Zn interstitial which leads to a carrier-mediated ferromagnetism while the ZnO:Pd system possesses no ferromagnetism.

PACS 31.15.E-, 75.50.Pp, 75.30Hx

## Introduction

Semiconductor metal oxides, with applications in the photoelectrochemical cells, diluted magnetic semiconductors (DMS), field effect transistors, and photoluminescence devices, have recently initiated dynamic research activities [1-3]. In particular, ZnO has a significant advantage for applications in optical [4] and spintronic [5] devices. As a result, doping ZnO with various elements has been a popular technique to manipulate and control ZnO's extrinsic properties for device applications [6]. Specially rare earth (RE)- and transition metal (TM)-doped ZnO systems exhibit interesting optical and magnetic properties, which do not exist in undoped ZnO. Optically, ZnO systems doped with RE ions have been intensively investigated as electroluminors with wide technological applications [7]. In RE-doped ZnO, the intra-ionic 4*f *transitions of RE ions form luminescent centers which generate narrow and intense emission lines [8]. While the enhancement in the optical absorption of TM-doped ZnO can transfer these materials to efficient photocatalysts [9].

Magnetically, the intrinsic magnetic moment, borne by the RE and TM ions, makes the RE and TM-doped ZnO systems to be potential diluted magnetic semiconductors with applications in spintronic devices. Over the past decade or so, a considerable amount of effort has been made on searching for ZnO-based DMSs with ferromagnetism at ambient. This goal is meant to be achieved by doping ZnO with mainly the first row TMs, such as Co, Mn, and Fe [10]. However, most recently, interesting magnetism has been observed in other metal oxides doped with the second row TMs, namely in Sn_2_O:Pd system [11]. This stimulated further search for possible ferromagnetism [12] and functional optical properties [13] in ZnO:Pd. The realization of magnetism in the ZnO:Pd system is motivated by a previous theoretical prediction [14] and experimental observation [15] of ferromagnetism in Pd clusters. In addition to systems containing TM ions, Eu-doped ZnO (ZnO:Eu) has also shown room temperature ferromagnetic ordering [16] which is partially caused by the high magnetic moment of the Eu ions. In this work, the structural and electronic properties of the ZnO:Pd and ZnO:Eu systems are investigated by a density functional approach. Furthermore, the effects of ZnO's two dominant point defects [17], Zn interstitial (Zn_I_) and O vacancy (V_O_), on the functional properties of these materials are studied. The results of theoretical investigations presented here will shed light on the origin of the functional properties of this relatively new family of materials.

### Computational details

*Ab initio *calculations were performed with a density functional theory-based DMol3 package developed by Accelrys [18,19]. Geometry optimization and partial density of states (PDOS) calculations were performed with "double-numeric plus polarization" (DNP) basis set while generalized gradient approximation (GGA) based on Perdew-Wang formalism was applied for correlation functional [20]. Real-space global cutoff radii were set for all elements at 5 Å. Since only valence electrons would affect the physical properties, the nuclei and core electrons were replaced by DFT semi-core pseudopotentials with a relativistic correction [21]. A Brillouin zone sampling was carried out by choosing the 2 × 2 × 2 *k*-point set using the Monkhorst-Park scheme with a grid spacing of approximately 0.04 Å^-1 ^between *k *points. The convergence thresholds for energy, Cartesian components of internal forces acting on the ions, and displacement were set to be 10^-5 ^eV/atom, 0.05 eV/Å, and 0.001 Å, respectively. A convergence testing was performed, first by increasing the *k *point mesh to 3 × 3 × 3; it was found that the total energy differs less by 10^-5 ^eV/atom. Then, the *k*-point mesh was fixed at 2 × 2 × 2, and the cutoff radii were set for all elements to be 5.5 Å. Once again, no significant change in the total energy was obtained. Thus, the results were well converged.

The formation enthalpy and bandgap for undoped ZnO was calculated to be -3.5 and 2.0 eV, respectively. The formation enthalpy is in good agreement with the experimental value of -3.64 eV [22]. However, the bandgap is underestimated by 1.4 eV which is attributed to the GGA intrinsic error. The calculated lattice constants for undoped and fully optimized ZnO were found to be 3.279 Å for *a *and 5.281 Å for *c*, which are in good agreement with the experimental data [23], overestimated by only 0.9% and 1.5%, respectively. The Zn-O bond lengths in the relaxed structure were 2.005 and 1.997 Å along the *c *direction and *ab *plane, respectively. In order to avoid the artificial hydrostatic stress in the doped structures, the lattice parameters were fixed to the calculated values of the undoped ZnO while only the internal atomic coordinates were relaxed.

To simulate the low concentrations of dopants in ZnO, a large supercell of 4*a × *4*a × *2*c *was adopted for calculations. The original supercell contained 64 Zn-O formula units. By introducing one dopant in the substitutional or interstitial site, the concentration of the dopants would be 1.4%. Having the periodic boundary conditions applied, the average separation of the dopant ions is 13.114 Å. This distance is large enough to avoid artificial interaction between the dopants. As a result, the calculations on this supercell will sufficiently resemble the experimental conditions of diluted dopant concentrations. The formation energy (*E*^f^) of the dopants or a cluster of dopants in ZnO's host lattice was calculated as follows:(1)

in which *E*^t^, *μ*, and *E*_F _represent total energy, chemical potential for respective elements, and Fermi energy, respectively. *μ*_Zn _and *μ*_M _are set to be the calculated energies of metallic Zn and the dopants (Pd or Eu) per element. *n *represents the number of Zn atoms removed from the supercell, which is zero in the case of the interstitial dopant and one for the substitutional dopant. *q *stands for the net number of electrons transferred from the defect to the conduction band. Since only neutral supercells were adapted for the calculations, *q *is zero for all configurations.

### The ZnO:Eu system

**Figure 1:**
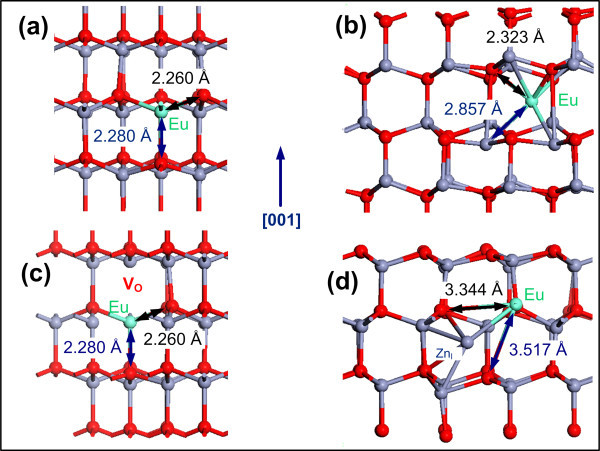
**The relaxed crystallographic structure of the ZnO:Eu systems**. Representing **(a) **ZnO:Eu_Zn_, **(b) **ZnO:Eu_I_, **(c) **ZnO:Eu_Zn _+ V_O_, **(d) **ZnO:Eu_Zn _+ Zn_I _systems. Red, blue, and gray balls represent the O, Zn, and Eu ions, respectively. For each system, the calculated length of the Eu bonds is presented along both the *c *direction (or [001]) and within the *ab *(basal) plane.

The incorporation mechanism of Eu ions in ZnO's host lattice was investigated by calculating the *E*^f ^of the substitutional Eu (Eu_Zn_) and interstitial Eu (Eu_I_) in the stochiometric ZnO as presented in Table 1. It was found that the *E*^f ^of Eu_Zn _is -2.391 eV while the *E*^f ^of Eu_I _is 1.429 eV. Such a large difference in *E*^f ^indicates that Eu ions favorably substitute Zn ions rather than taking the interstitial sites of the ZnO lattice. The local geometry of the Eu_Zn _and Eu_I _is presented in (a) and (b). Figure 1a shows that the length of Eu-O bond along *c *direction (*ab *plane) has increased to 2.280 Å (2.260 Å) in the ZnO:Eu_Zn _system. The increase of the bond lengths is approximately equivalent to an expansion of 14% along the *c *direction and 13% within the *ab *plane with respect to the Zn-O bond length in an undoped ZnO. On the other hand, in the ZnO:Eu_I _system, in which Eu_I _binds to three Zn and three O ions, the length of the Eu-O and Eu-Zn bonds were found to be 2.323 and 2.857 Å comprising an expansion of 16% and 24%, respectively compared to the unrelaxed structure. In the ZnO:Eu_Zn _system, although the length of the Eu bonds is also substantially expanded, the expansion is much smaller than that in the ZnO:Eu_I _system. As a result, the ZnO:Eu_Zn _system reaches the structural stability with less lattice distortion.

**Table 1:** The *E*^f^, Eu's spin number, and the magnetic ground state of the ZnO:Eu_Zn_, ZnO:Eu_I_, ZnO:Eu_Zn _+ V_O _and ZnO:Eu_Zn _+ Zn_I _are presented.

Configuration	***E***^**f **^**(eV)**	Eu's spin ([ℏ]/2)	Magnetic ground state
ZnO:Eu_Zn_	-2.391	6.800	Paramagnetic
ZnO:Eu_I_	1.429	6.859	Paramagnetic
ZnO:Eu_Zn _+ V_O_	1.772	6.907	Paramagnetic
ZnO:Eu_Zn_:Z_I_	2.776	6.879	Ferromagnetic

Next, the *E*^f ^of Eu_Zn _in the nonstochiometric ZnO was studied by considering two distinct situations that lead to Zn excess: first by including a V_O _in the ZnO:Eu_Zn _+ V_O _system and then by including a Zn_I _in the ZnO:Eu_Zn _+ Zn_I _system. The *E*^f ^of the Eu_Zn _+ V_O _and Eu_Zn _+ Zn_I _complexes in the ZnO lattice was found to be 1.772 and 2.776 eV, respectively. The local geometry of the Eu ion in the ZnO:Eu_Zn _+ V_O _and ZnO:Eu_I _+ V_O _are presented in Figure 1c, d respectively. In the ZnO:Eu_Zn_:V_O _system, the length of the Eu-O bond is 2.280 along the *c *direction and 2.260 within the *ab *plane, which is identical to the bond length in the ZnO:Eu_Zn _system without V_O_. However, in the ZnO:Eu_Zn_+Zn_I _system, the length of the Eu-O bond expanded to 3.517 Å along the *c *direction and 3.344 Å within the *ab *plane. Such an expansion corresponds to a 75% and 67% increase in the length of the Eu-O bonds along the *c *direction and within the *ab *plane, respectively compared to the unrelaxed structure. As a result, the Eu_Zn _+ Zn_I _complex has the highest *E*^f ^and lattice distortion.

**Figure 2:**
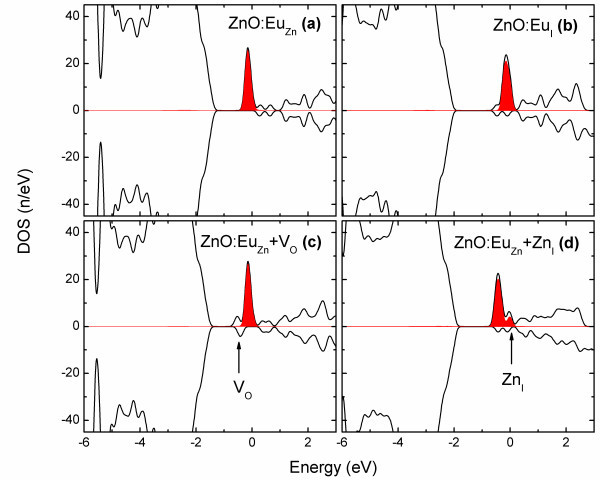
**Total and partial DOS of the ZnO:Eu systems**. Representing **(a) **ZnO:Eu_Zn_, **(b) **ZnO:Eu_I_, **(c) **ZnO:Eu_Zn _+ V_O_, **(d) **ZnO:Eu_Zn _+ Zn_I _systems. The solid black lines and the red-shaded areas represent the total and Eu's partial 4*f *states, respectively. Also, energy is represented with respect to the Fermi level.

To investigate the electronic properties of the ZnO:Eu systems, the total and Eu's 4*f *partial density of states (DOS) of all configurations were calculated and presented in Figure 2. A general feature of the Eu's 4*f *states in all configurations is that Eu's 4*f *states are localized in a narrow impurity band of the width of approximately 1 eV, which is located just below the Fermi level. Such localization of the 4*f *states indicates that 4*f *electrons are not affected by the local crystal environment. This point is further reinforced by the Eu's magnetization as presented in Table 1. The spin number (*S*) of the Eu ions in all configurations is approximately 6.9, very close the spin number of free Eu atoms which indicates the infinitesimal hybridization of Eu's *f *orbitals with other orbitals in the host crystals. According to Figure 2a, b, in stochiometric systems, ZnO:Eu_Zn _and ZnO:Eu_I_, there are minor electronic states available at the Fermi level resulting in limited mobile carriers in those systems. However, this amount of carriers is not sufficient to establish carrier-mediated magnetism in the stochiometric ZnO:Eu systems, and these systems remain paramagnetic [24]. By introducing V_O_, in the ZnO:Eu_Zn _+ V_O _system, the V_O_'s impurity states appear below Eu's 4*f *states as shown in Figure 2c. Thus, V_O _does not enhance the carrier concentration in the ZnO:Eu_Zn _+ V_O _system either. As shown in Figure 2d in the ZnO:Eu_Zn _+ Zn_I _system, Zn_I_'s 4*s *states appear in a small peak at the Fermi level, partially hybridizing with Eu's 4*f *states and introducing further carriers at the Fermi level. To investigate the possibility of ferromagnetic coupling in the defective systems, two substitutional Eu ions were located in the supercells. Then the *E*^f ^of each system was calculated once for ferromagnetic magnetic alignment (*E*^FM^) and once again for antiferromagnetic magnetic alignment (*E*^AFM^) of the Eu ions. Finally, Δ*E *is defined to be *E*^AFM ^- *E*^FM ^which is an indicator of ferromagnetic phase stabilization. For Eu ions separated by approximately 3.4 Å (nearest possible distance), Δ*E *was found to be 21 meV for the ZnO:Eu + Zn_I _system and 3 meV for the ZnO:Eu + V_O _system. However, for both systems, the Δ*E *vanishes when the separation between the Eu ions increases to approximately 6 Å. This trend in Δ*E *indicates that Zn_I _induces short range ferromagnetic coupling in the ZnO:Eu system.

### The ZnO:Pd system

**Figure 3:**
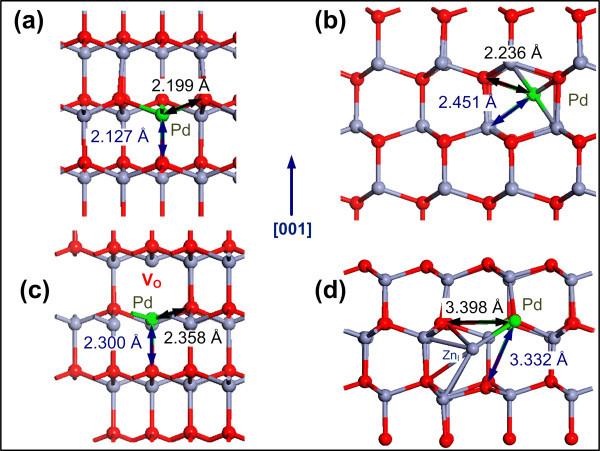
**The relaxed crystallographic structure of the ZnO:Pd systems**. Representing **(a) **ZnO:Pd_Zn_, **(b) **ZnO:Pd_I_, **(c) **ZnO:Pd_Zn _+ V_O_, **(d) **ZnO:Pd_Zn _+ Zn_I _systems. Red, blue, and gray balls represent the O, Zn, and Pd ions, respectively. For each system, the calculated length of the Eu bonds is presented along both the *c *direction (or [001]) and within the *ab *(basal) plane.

In the ideal ZnO lattice, the length of the O-Zn bond within the basal plane is 1.997 Å. The radius of O^-2 ^being 1.40 Å, leaves enough room for Pd^+2 ^with an atomic radius of 0.64 Å to substitute the Zn^+2 ^ion without significant lattice distortion to create a Pd_Zn_. Alternatively, Pd^+2 ^can fit in the octahedral interstitial site which is located in the interstitial channel along *c *axis. In addition to the octahedral interstitial site, there is a tetrahedral interstitial site in ZnO which has a Zn^+2 ^ion and an O^-2 ^ion as nearest-neighbor atoms, at a distance of about 1.67 Å, (0.833 times the Zn-O bond length along the *c *axis). Thus, a Pd ion cannot be placed at this site without severe geometric constraints. In order to determine the preferred site of the Pd ion in the ZnO lattice, the *E*^f ^of Pd for both the ZnO:Pd_I _and ZnO:Pd_Zn _systems were calculated. It was found that the *E*^f ^of Pd_Zn _and PdI were 0.776 and 1.612 eV. Such a difference results in high concentration of Pd_Zn _over the Pd_I _in thermal equilibrium condition in the ZnO:Pd system. Such a finding is in agreement with the reported experimental data that Pd^+2 ^tends to substitute Zn^+2 ^in ZnO [13]. Figure 3a, b show the local geometry of Pd_Zn _and Pd_I _in ZnO. For the ZnO:Pd_Zn _system, the Pd-O is 2.127 and 2.199 Å along *c *direction and within the *ab *plane, respectively with approximate expansions of 6% and 10%, respectively compared to the unrelaxed structure. For the ZnO:Pd_I _system, the Pd-O and Pd-Zn bonds have increased to 2.236 and 2.451 Å, respectively with the expansions of 11% and 6% with respect to the unrelaxed structure. Similar to the ZnO:Eu system, in the ZnO:Pd system, Pd_Zn _has a lower *E*^f ^and causes less lattice distortion. Electronically, a Mullikan population analysis indicated that both Pd_Zn _and Pd_I _are isovalent to the Zn ions, transferring two electrons to neighboring O ions. This implies that Pd's 4*d *shell remains fully occupied, thus the Pd ions are not magnetized in the ZnO host lattice which is reflected in zero magnetization of Pd ions in all configurations as presented in Table 2. As a result, the stochiometric ZnO:Pd system is nonmagnetic.

**Table 2:** The *E*^f^, Pd's spin number, and the magnetic ground state of the ZnO:Pd_Zn_, ZnO:Pd_I_, ZnO:Pd_Zn _+ V_O_, and ZnO:Pd_Zn _+ Zn_I _are presented.

System	***E***^**f **^**(eV)**	Pd's spin ([ℏ]/2)	Magnetic ground state
ZnO:Pd_Zn_	0.776	0.000	Nonmagnetic
ZnO:Pd_I_	1.612	0.000	Nonmagnetic
ZnO:Pd_Zn _+ V_O_	6.289	0.000	Nonmagnetic
ZnO:Pd_Zn_:Z_I_	3.831	0.000	Nonmagnetic

Similar to the previous section, the effect of V_O _and Zn_I _on the ZnO:Pd systems was investigated by calculating the *E*^f ^of the Pd_Zn _+ V_O _and Pd_Zn _+ Zn_I _complexes in ZnO, which was found to be 6.289 and 3.831 eV, respectively. In the ZnO:Pd_Zn _+ V_O _system, the Pd-O bond is 2.300 and 2.358 Å along the *c *direction and the *ab *plane, respectively, having 15% (18%) expansion in bond lengths with respect to the unrelaxed structure. In the ZnO:Pd_Zn _+ Zn_I _system, the lengths of the Pd-O bond along the *c *direction and within the *ab *plane are 3.332 and 3.398 Å, respectively which comprise an expansion of 66% along the *c *direction and an expansion of 70% within the *ab *plane compared to the unrelaxed structure.

**Figure 4:**
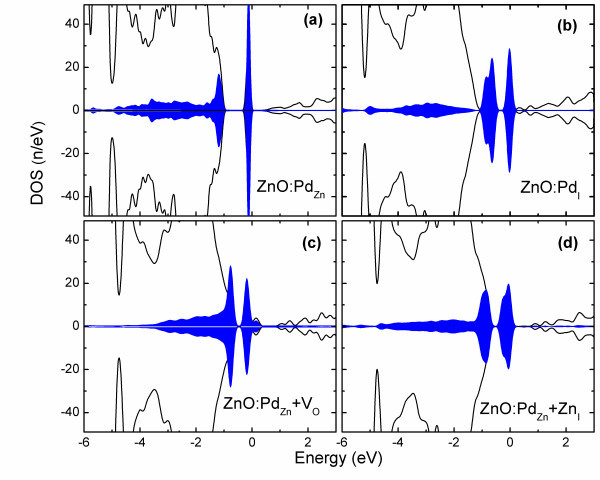
**Total and partial density of states of the ZnO:Pd systems**. Representing **(a) **ZnO:Pd_Zn_, **(b) **the ZnO:Pd_I_, **(c) **ZnO:Pd_Zn _+ V_O_, **(d) **ZnO:Pd_Zn _+ Zn_I _systems. The solid black lines and the blue-shaded areas represent the total and Pd's partial 4*d *states, respectively. For clarity, the Pd's *d *states are scaled by a factor of five. Also, energy is represented with respect to the Fermi level.

To investigate the electronic properties of the ZnO:Pd systems, the total and partial density of states of both systems were calculated and presented in Figure 4. As in Figure 4a, the 4*d *states of Pd_Zn _and O's 2*p *states hybridize and form bonding (*t*^b^) states in the valence band. The antibonding 4*d *states with *e *symmetry are located above the *t*^b ^states, separated by a gap of approximately 1 eV from the valance band maximum and positioned just below the Fermi Level. The position of the Pd_Zn _antibonding states with respect to the Fermi level indicates an *n*-type behavior of the ZnO:Pd_Zn_. Notably, the 4*d *states are completely degenerate for spin-up and spin-down states with no exchange splitting. This implies that Pd_Zn _does not induce any magnetization. For Pd_I_, according to Figure 4b, the 4*d *states of Pd_I _are split into bonding and antibonding states. The bonding states with the *t*^b ^symmetry hybridize with O's 2*p *orbitals along the valance band. The antibonding states with *e *symmetry are divided into three peaks in the fundamental bandgap region. This is the major difference of the DOS of the Pd_I _and Pd_Zn_, which may be caused by the different crystal fields experienced by each site. The closely located peaks in the bandgap region results in the high optical activity of the ZnO:Pd_I _system, in particular, they may be the mechanism behind the observed red shift in the photoluminescence spectrum in the ZnO:Pd system [13]. The position of the *e *states from the Fermi level and the absence of exchange splitting indicate that Pd_I _is an *n*-type dopant with no magnetization. In nonstochiometric systems, ZnO:Pd_Zn _+ V_O _and ZnO:Pd_Zn _+ Zn_I_, the Pd's 4*d *states, as shown in Figure 4c, d, are distributed in a similar pattern to the ZnO:Pd_Zn_. It exhibits the Pd's spin-up and spin-down states which are fully degenerate, indicating the absence of any magnetization per Pd ion. Therefore, these systems are nonmagnetic even when V_O _and Zn_I _exist in the ZnO:Pd system.

## Conclusion

The structural and electronic properties of the ZnO:Eu and ZnO:Pd systems were investigated by *ab initio *techniques. It was found that both Eu and Pd ions substitute Zn sites in the ZnO host lattice favorably. With Zn excess in the ZnO:Eu system, the carrier-mediated ferromagnetism can be induced by Zn_I_. In the ZnO:Pd system, the Pd ions prefer to substitute Zn sites, forming substitutional Pd. Additionally, the Pd ions are isovalent to Zn ions and, consequently, their 4*d *shell remains fully occupied with no magnetization per Pd ion.

## Abbreviations

DFT: density functional theory; DMS: diluted magnetic semiconductors; DNP: double numerical polarized; DOS: density of states; GGA: generalized gradient approximation; PDOS: partial density of states.

## Competing interests

The authors declare that they have no competing interests.

## Authors' contributions

MHNA performed the computational work and drafted the manuscript. YBZ, RKZ, SPR, and SL jointly developed the model and co-drafted the manuscript. All authors read and approved the final manuscript.
